# Supplementary material for: Foxn1-β5t transcriptional axis controls CD8+ T-cell production in the thymus
Source: Nat Commun. 2017 Feb 8;8:14419. doi: 10.1038/ncomms14419 (PMC5309848; doi:10.1038/ncomms14419)
Supplement: Supplementary Information — Supplementary Figures and Supplementary Table [file ncomms14419-s1.pdf]

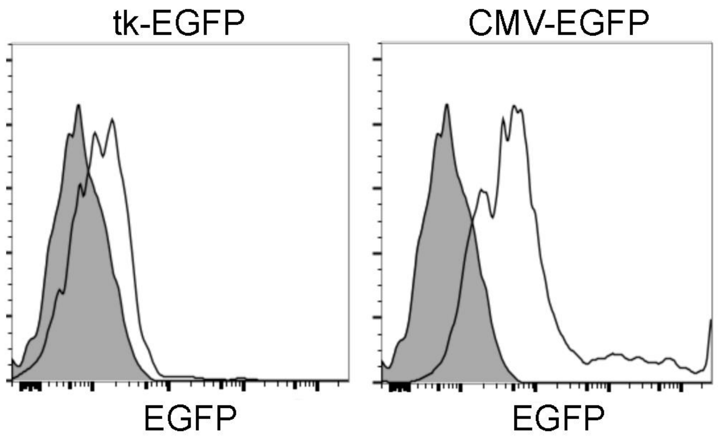

Supplementary Figure 1. EGFP expression by two different promoters. Representative profiles (n = 3) of EGFP expression in HEK293T cells transfected with EGFP-expressing plasmid driven by herpes simplex virus thymidine kinase gene promoter (tk-EGFP, left panel) or cytomegalovirus immediate early gene promoter (CMV-EGFP, right panel). Shaded histograms indicate EGFP expression in control cells.

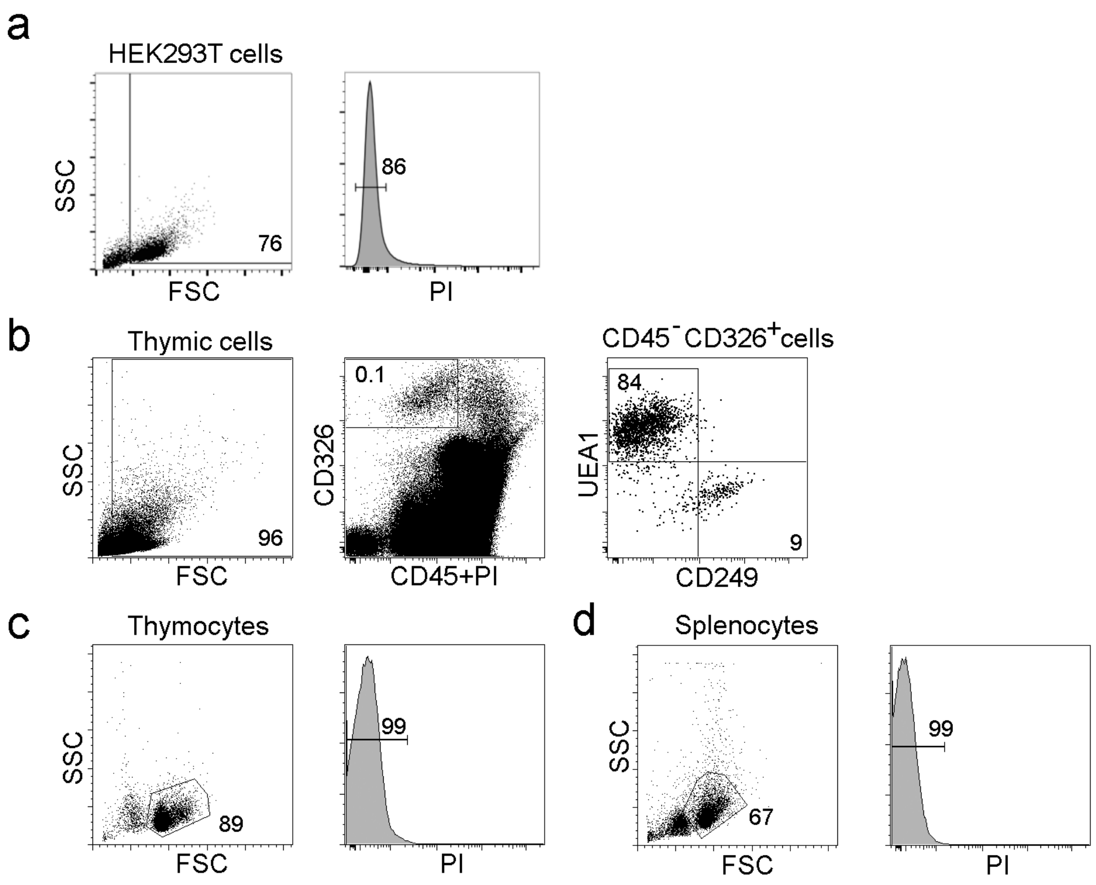

Supplementary Figure 2. Flow cytometry strategies. Dot plots and histograms show the representative flow cytometry profiles for the gating for (**a**) viable HEK293T cells, (**b**) liberase-digested viable $CD249^{+}UEA1^{-}CD45^{-}CD326^{+}$ cortical thymic epithelial cells and $CD249^{-}UEA1^{+}CD45^{-}CD326^{+}$ medullary thymic epithelial cells, (**c**) viable thymocytes, and (**d**) viable splenocytes. FSC, forward scatter; SSC, side scatter; PI, propidium iodide.

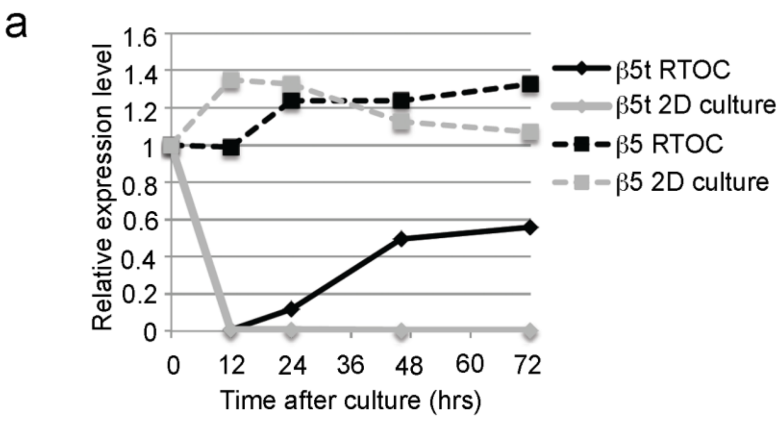

b

Gene Name	Accession number	E15.5	E14.5
5830406J20Rik (β5t)	NM_175204	19.82	23.11
Foxn1	NM_008238	6.09	6.50
Hey1	NM_010423	40.79	74.08
1700021K02Rik (Spatial)	NM_023064	18.21	20.01

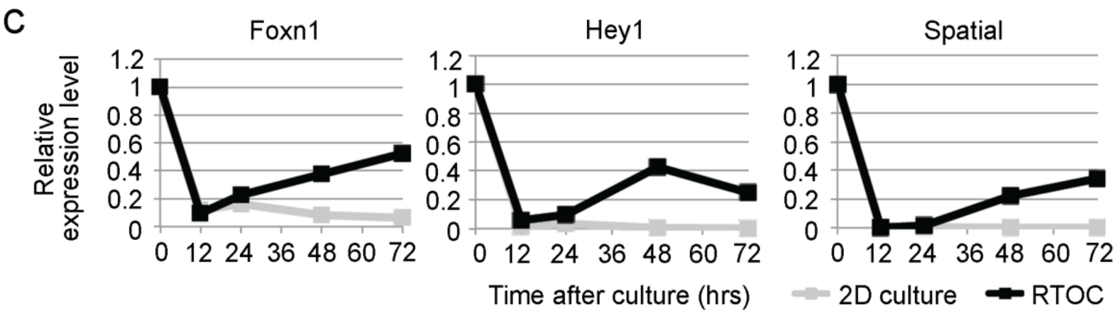

Supplementary Figure 3. Kinetics of gene expression in RTOC and 2D culture. (**a**) Relative mRNA expression of *β5t* (solid lines) and *β5* (dashed lines) in thymic stromal cells during RTOC (black lines) and 2D culture (gray lines). The expression level at 0 hr (just after disperse treatment) was set as 1. (**b**) Ratio of *β5t*, *Foxn1*, *Hey1*, and *Spatial* mRNA expression in RTOC versus 2D culture in microarray analysis of thymic stromal cells. (**c**) Relative mRNA expression of *Foxn1*, *Hey1*, and *Spatial* in thymic stromal cells during RTOC (black lines) and 2D culture (gray lines).

	sequence	location	homology to on-target	score
on-target	GGAGGACGCTGTGGAGAAGCTGG	chr14, β5t, 5'genomic region	100%	
off-target1	AGAGGAAGCTGTGGAGAAGCAGG	chr14, Sacs, intron2	90%	4.6
off-target2	GGGGGAAGCTGTGGAGAAGCTGG	chr2, Ryr3, intron73	90%	4.0
off-target3	GGGGGACCCAGTGGAGAAGCTGG	chr18, Zfp608, 3'genomic region	85%	2.4
off-target4	AGAGGACGGAGTGGAGAAGCAAG	chr4, Padi4, intron10	85%	1.5
off-target5	AGTGGTCGCTGTGGAGAAGCAGG	chr17, Cyp1b1, intron1	85%	1.5
off-target6	GGTGAACGCTGAGGAGAAGCAGG	chr4, Runx3, 5'genomic region	85%	1.3
off-target7	GCAGGACACTGTGGAGCAGCAGG	chr8, Agpat5, 5'genomic region	85%	1.3
off-target8	GGAGAACACTGGGGAGAAGCCAG	chr5, 2900026A02Rik, exon10	85%	1.3
off-target9	GGAGGACCCAGAGGAGAAGCTGG	chr12, Nrac, 5'genomic region	85%	1.1
off-target10	TGAGGTTGCTGTGGAGAAGCAGG	chr1, Esrrg, 5'genomic region	85%	1.1
off-target11	GGAGAACCCTGTGGAGAACCCGG	chr7, 9030624J02Rik, intron9	85%	1.0
off-target12	GGCGGAAGCTGTGGAGAAGGAGG	chr8, Maf, 5'genomic region	85%	1.0
off-target13	GGGGCACACAGTGGAGAAGCCAG	chr15, Prlr, 5'genomic region	80%	1.3
off-target14	TGAGAACCCAGTGGAGAAGCAGG	chr2, Ssfa2, 3'genimoc region	80%	1.3
off-target15	TCAAGAGGCTGTGGAGAAGCAAG	chr1, Gaptc2, 3'genomic region	80%	0.9

```
on-target     GGAGGACGCTGTGGAGAAGCTGG
              ******* ** ************
mouse genome  GGAGGACACTATGGAGAAGCTGG

off-target1   AGAGGAAGCTGTGGAGAAGCAGG
              ***********************
mouse genome  AGAGGAAGCTGTGGAGAAGCAGG

off-target2   GGGGGAAGCTGTGGAGAAGCTGG
              ***********************
mouse genome  GGGGGAAGCTGTGGAGAAGCTGG

off-target3   GGGGGACCCAGTGGAGAAGCTGG
              ***********************
mouse genome  GGGGGACCCAGTGGAGAAGCTGG

off-target4   AGAGGACGGAGTGGAGAAGCAAG
              ***********************
mouse genome  AGAGGACGGAGTGGAGAAGCAAG

off-target5   AGTGGTCGCTGTGGAGAAGCAGG
              ***********************
mouse genome  AGTGGTCGCTGTGGAGAAGCAGG

off-target6   GGTGAACGCTGAGGAGAAGCAGG
              ***********************
mouse genome  GGTGAACGCTGAGGAGAAGCAGG

off-target7   GCAGGACACTGTGGAGCAGCAGG
              ***********************
mouse genome  GCAGGACACTGTGGAGCAGCAGG

off-target8   GGAGAACACTGGGGAGAAGCCAG
              ***********************
mouse genome  GGAGAACACTGGGGAGAAGCCAG

off-target9   GGAGGACCCAGAGGAGAAGCTGG
              ***********************
mouse genome  GGAGGACCCAGAGGAGAAGCTGG

off-target10  TGAGGTTGCTGTGGAGAAGCAGG
              ***********************
mouse genome  TGAGGTTGCTGTGGAGAAGCAGG

off-target11  GGAGAACCCTGTGGAGAACCCGG
              ***********************
mouse genome  GGAGAACCCTGTGGAGAACCCGG

off-target12  GGCGGAAGCTGTGGAGAAGGAGG
              ***********************
mouse genome  GGCGGAAGCTGTGGAGAAGGAGG

off-target13  GGGGCACACAGTGGAGAAGCCAG
              ***********************
mouse genome  GGGGCACACAGTGGAGAAGCCAG

off-target14  TGAGAACCCAGTGGAGAAGCAGG
              ***********************
mouse genome  TGAGAACCCAGTGGAGAAGCAGG

off-target15  TCAAGAGGCTGTGGAGAAGCAAG
              ***********************
mouse genome  TCAAGAGGCTGTGGAGAAGCAAG
```

Supplementary Figure 4. No detection of off-target mutations in site #13 mutant mice. On-target site #13 and fifteen off-target sites with the high sequence match (80% to 90% of the similarity excluding the protospacer adjacent motif) to the on-target site (top) were sequenced in the genome of mutant mice (bottom). Underlines indicate the protospacer adjacent motif sequence. No off-target mutations were detected in our site #13 mutant mice.

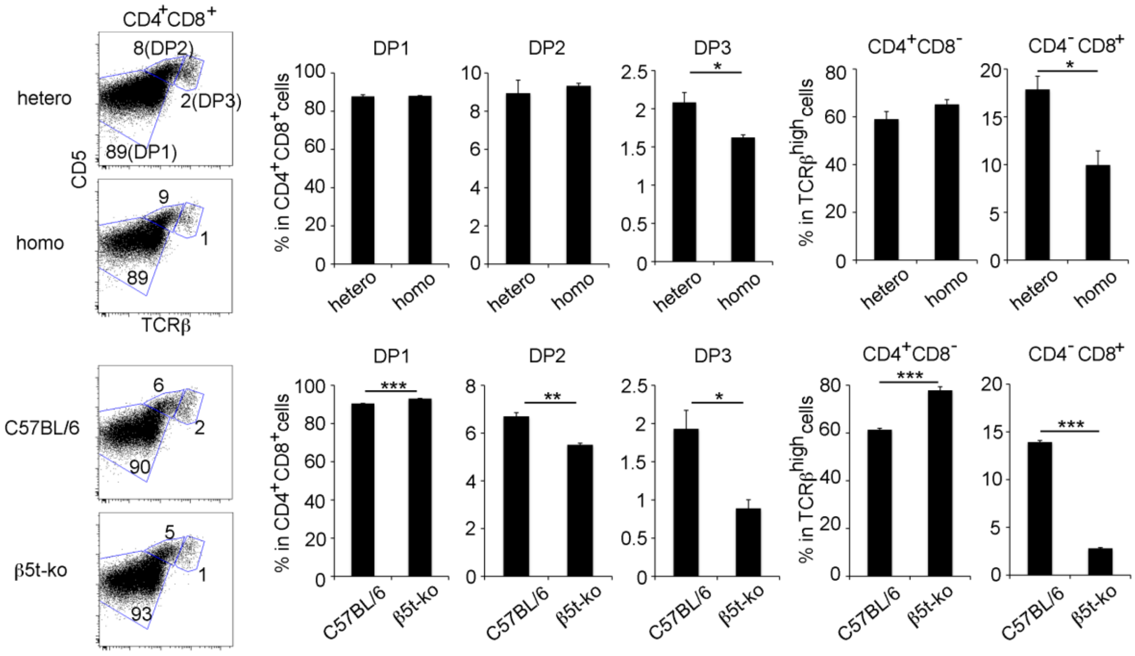

Supplementary Figure 5. Diminished DP3 thymocytes in site #13 mutant mice. Flow cytometric analysis of thymocytes from 5-week-old mice. Shown are dot plots for TCRβ and CD5 expression in $CD4^{+}CD8^{+}$ cells. Boxes indicate DP1 (TCR^{low} $CD5^{low}$), DP2 ($TCR^{intermediate}$ $CD5^{high}$) and DP3 (TCR^{high} $CD5^{intermediate}$) subpopulations. Numbers in dot plots indicate frequency of cells within indicated area. Bar graphs show the frequency (means and s.e.m., n = 3) of indicated thymocyte populations. *, P<0.05; **, P<0.01; ***, P<0.001. Statistic analyses were performed by student's *t*-test.

Supplementary Table 1. Nucleotide sequences of site #8 and site #13 in different species. Letters in red indicate the Foxn1-binding invariant core ACGC tetranucleotide.

	site #8	site #13
Mouse	GCTTACGCTTT	GAGGACGCTGT
Human	TAACACTCACC	AGGGACGCTAC
Chimpanzee	GCTGAGGCAGG	AGGGACGCTAC
Dog	GTATATCATCT	AGAGACGCTAA
Rat	GCATACTCTTT	GAGGACGCTGT
Bat	TATCACTTACC	AAGGACGCTAA
Elephant	CCTACTCATAA	AGGGACGCTAG
Horse	GGTCAAAGGGT	AGGGACGCTAA
